# Comparison of terminally ill cancer- vs. non-cancer patients in specialized palliative home care in Germany – a single service analysis

**DOI:** 10.1186/s12904-015-0033-z

**Published:** 2015-07-25

**Authors:** Stephanie Stiel, Maria Heckel, Andreas Seifert, Tobias Frauendorf, Roland Martin Hanke, Christoph Ostgathe

**Affiliations:** Department of Palliative Medicine, Comprehensive Cancer Center CCC Erlangen-EMN, University Hospital Erlangen, Friedrich-Alexander-Universität Erlangen-Nürnberg, Krankenhausstraße 12, 91054 Erlangen, Germany; Palliative Care Team (PCT) Team, Fürth, Germany; Innovation Incubator, Leuphana University of Lüneburg, Lüneburg, Germany

**Keywords:** Palliative care, Home care, Non-cancer, Non-malignant, Spezialisierte ambulante palliativversorgung

## Abstract

**Background:**

Palliative care (PC) is no longer offered with preference to cancer patients (CA), but also to patients with non-malignant, progressive diseases. Taking current death statistics into account, PC in Europe will face a growing number of patients dying from non-cancer diseases (NCA). More insights into specialized palliative home care (SPHC) in NCAs are needed.

**Methods:**

Retrospective analysis and group comparisons between CAs and NCAs of anonymous data of all patients cared for between December 2009 and June 2012 by one SPHC team in Germany. Patient-, disease- and care-related data are documented in clinical routine by specialized PC physicians and nurses in the Information System Palliative Care 3.0 ® (ISPC®).

**Results:**

Overall, 502 patients were cared for by the SPHC team; from 387 patients comprehensive data sets were documented. These 387 data sets (CA: *N* = 300, 77.5 % and NCA: *N* = 87, 22.5 %) are used for further analysis here. NCAs were significantly older (81 vs. 73 years; *p* < .001), than CAs and most often suffered from diseases of the nervous system (40 %). They needed significantly more assistance with defecation (87 vs. 74 %; *p* < .001) and urination (47 vs. 29 %; *p* < .001) and were more often affected from impaired vigilance (30 vs. 11 %; *p* < .001) than CAs. A by trend higher proportion of NCAs died within one day after admission to palliative home care (12 vs. 5 %; *p* < .05) and a smaller proportion was re-admitted to hospital during home care (6 vs. 20 %; *p* < .001). NCAs died predominantly in nursing homes (50 vs. 20 %; *p* < .001).

**Conclusions:**

Although the proportion of NCAs was relatively high in this study, the access to PC services seems to takes place late in the disease trajectory, as demonstrated by the lower survival rate for NCAs. Nevertheless, the results show, that NCAs PC needs are as complex and intense as in CAs.

## Background

During the last decades, hospice and PC has dynamically evolved in Europe [1]. One central development in Germany is the growing number of in- and outpatient palliative and hospice care services [2]. Parallel to this, first reimbursement models for inpatient and home PC have emerged. Furthermore, palliative medicine now plays a growing role in education for medical students, nurses and other healthcare professionals.

The PC approach was primarily developed to support incurable CA patients. Over the last decades, however, there has been growing evidence that also patients with non-malignant, progressive diseases are in need and may benefit from PC [3–5]. This is underpinned by the fact that all “patients […] facing the problem associated with life-threatening illness” independent of their underlying primary disease are included in the World Health Organization’s (WHO’s) definition of PC [6]. However, in Germany - as in many other countries - barriers to palliative and hospice care for NCA patients still exist [7]. In fact the percentage of NCA patients receiving inpatient PC, for example, is rising while the overall percentage, however, is still disproportionately low [8, 9]. Taking projections of statistics on death and current findings into account, palliative and hospice care in Europe will face a growing number of patients suffering and dying from non-malignant diseases [10–12].

Despite this protracted evolution, palliative home care for patients with NCA diseases is also increasingly deserving of attention [13]. In 2007, a national bill passed (§37b social security statutes book V), securing Germans with life limiting advanced diseases and complex needs the right to receive specialised PC at home [14]. Since then regional specialised palliative home care services (SPHC) have evolved throughout the country. The requirements for SPHC providers are uniform concerning staffing, quality, education and standards for adequate care provision. PC teams are composed of physicians and nurses supported by other professionals and therapists if necessary [15]. SPHC teams treat patients in their own homes, in nursing homes or hospices alongside (not replacing) their primary care doctors and nursing services. SPHC support (24/7) includes symptom control, counselling for patients and their relatives, professional and informal caregivers, and general practitioners, as well as organization, planning and coordination of involved services, interventions in medical or psycho-social crises, support in decision-making procedures and management of ethical conflicts. All that support is meant to enable patients to stay in their familiar environment until death, if possible.

The involvement of SPHC can be prescribed by a hospital physician or a practitioner. SPHC starts (almost) always, if formally prescribed, for at least the first 7 days and continues if the health insurance approves reimbursement of the costs [16]. The necessity for SPHC has to be re-evaluated on a regular basis. The main eligibility criteria for the SPHC service are i) advanced progressive disease, ii) at least two of seven complex symptom cluster (pain, respiratory-cardiac, neurologic-psychiatric-psychological, gastrointestinal, urogenital, ulcerated wounds or tumors, others) and iii) a necessity for 24/7 on-call service, avoidance for hospital readmission, expected crisis intervention or symptom management. The SPHC service is financed from a case compensation independent from care expenses.

To date limited data are available regarding the daily work of SPHC teams in Germany and, moreover, little specific data on SPHC for terminally ill patients suffering from non-malignant diseases who may have differing needs from CA patients receiving PC exist [5]. There are only few studies and comments focussed on the treatment of specific aspects [15] and the problems, chances and expectations of general practitioners cooperating with SPHC teams [16, 17]. One study on SPHC teams in Bavaria has taken place in 2013 and allows for broader information on organisation, performance and its effect on patients and their relatives [18]. Therefore, this single institution analysis investigates SPHC in Germany delivered for NCA and CA patients. Research on the access to and utilization of PC services is highly important in particular with respect to NCA patients who often have similar symptoms and needs as CA patients but seem to be underrepresented in PC services.

### Study aim

The authors aim to answer the question whether terminally ill patients suffering from non-malignant versus malignant diseases receiving specialized palliative home care do have comparable demographic characteristics and PC needs or whether they form different patient populations.

## Methods

### Study setting

As a well-established regional team, the SPHC Fürth (Bavaria) was approached to cooperate for this study. The team started in 2009 and consists of six physicians and seven nurses working both in part or full time. All physicians of this single service regularly work in their private practice as family physicians or specialists and provide an on-call service for this SPHC team. The nurses work for the SPHC team around the clock. All team members are planned and organized so that each group of two physicians and nurses are available during the daytime from 08.00 a.m. until 20.00 p.m.. During the night between 20.00 p.m. and 08.00 a.m., a single physician and a nurse perform on-call duty.

### Data sets and study variables

Since its establishment in 2009, patient-, disease- and care-related data such as gender, age, marital status, living situation, residential area (urban vs. rural), primary diagnosis, nursing care level, advance directive, symptoms and problems and characteristics, number of visits and duration of SPHC, re-admittance to hospital and place of death are documented by specialized PC physicians and nurses.

Symptoms were assessed via a symptom- and problem checklist analogue to symptom- and problem checklist from the Hospice and Palliative care Evaluation (HOPE) [16] and were scaled as follows: 0 = none, 1 = mild, 2 = moderate, 3 = severe. Additional nursing care problems such as need for assistance with defecation and bladder function or deterioration of vigilance were added in the documentation of the SPHC team.

For data management, the Information System Palliative Care 3.0 ® (ISPC®), developed by Smart-Q Softwaresysteme GmbH, was used. The ISPC® database was adapted continuously during the first years according to the professionals’ documentation needs. As a result, some additional information relevant for their daily clinical practice was integrated into the initial database, so that several items were not documented during the whole period of the study analysis chosen here.

For this investigation, datasets from ISPC® were extracted to Microsoft Excel 2010 and tested for plausibility. Detected coding inconsistencies and missing data were checked and validated in the original, electronic ISPC® patient chart and added to the electronic database.

For this analysis no additional data collection, patient involvement or study measures took place. The study was approved by the local Ethics Committee (Ethik-Kommission der Friedrich-Alexander- Universität Erlangen-Nürnberg). After careful consideration of the selected study method of a retrospective data analysis, the ethics committee confirmed that no written informed consent had to be obtained from the patients and waived the requirement for informed consent before study application.

### Data analyses and statistics

This retrospective analysis used fully anonymous data of all patients cared for between December 2009 and June 2012. SPHC team members entered the data into ISPC®, an on-going data basis used for clinical routine practice, and a medical student finally extracted these data to SPSS.

The program IBM SPSS Statistics 21.0 (IBM Corporation, 1 New Orchard Road, Armonk, New York, United States) for Windows was applied for statistical analysis. Descriptive analysis of the data was performed.

To further investigate group differences between CA vs. NCA patients, T-Test in ratio or interval scaled variables or Chi^2^-Tests and Fisher’s Exact Tests for low given N in nominal or ordinal data in 2x2 contingence tables (two-tailed *p* < 0.01) were calculated. Additionally, logistic regression analyses were performed to test whether group differences in patient age between the NCA and CA group could explain other group differences.

## Results

### Study sample

Between December 2009 and June 2012, 502 patients were cared for by the SPHC team. Due to the formation and foundation of the SPHC team, in the beginning of their work documentation was based on non-systematic hand-written notes. The chronological first 115 data sets were not suitable for any data analysis and were therefore excluded. The development of a systematic documentation system became work-in-progress in the following time. More comprehensive and systematic data are available from 387 patients between October 2010 and June 2012 and were used here for further analysis. Due to the very low validity and range of data of the first 115 data sets, no comparison of patient characteristics between the data sets that were used and the excluded data sets was possible. The remaining 387 data sets include two independent subgroups of CA (*N* = 300, 77.5 %) and NCA (*N* = 87, 22.5 %) patients and are used for further analysis here.

### Demographic data

NCAs were tested significantly older (81 ± 10 years) than CAs (73 ± 12 years) with an almost equal proportion of female gender (58 vs. 55 %). Considering the individual marital status, NCA patients were significantly less often married (25 vs. 48 %), but more often widowed (38 vs. 23 %) than CA patients. According to this finding, NCA patients were living less often with relatives (30 vs. 53 %) or alone (7 vs. 18 %), but more often in nursing homes (55 vs. 22 %). (see Table 1). The significant group difference in marital status is controlled by the patients’ age in logistic regression (*p* = .165).

**Table 1:** Group differences between non-cancer (*N* = 87) vs. cancer patients (*N* = 300) in demographic data

		Non-cancer (*N* = 87)	Cancer (*N* = 300)	Level of significance
Age		81 ± 10 years	73 ± 12 years	*p* < .001
Female gender		58 %	55 %	*p* = .714
Marital status	Married	22 (25 %)	143 (48 %)	*p* < .001
	Widowed	33 (38 %)	69 (23 %)	
	Divorced	6 (7 %)	19 (6 %)	
	Single	2 (2 %)	16 (5 %)	
	Missing Data	24 (28 %)	53 (18 %)	
Living situation	With relatives	26 (30 %)	160 (53 %)	*p* < .001
	Alone	6 (7 %)	54 (18 %)	
	Nursing Home	48 (55 %)	67 (22 %)	
	Hospice	0 (0 %)	8 (3 %)	
	Missing Data	7 (8 %)	11 (4 %)	
Residential area	Urban	41 (47 %)	156 (52 %)	*p* = .466
	Rural	46 (53 %)	144 (48 %)	
Advance directive		36 (41 %)	145 (48 %)	*p* = .274

At the time of admission into SPHC care, NCA patients primarily wished to experience relief from suffering and symptom management (85 %), no re-admission to hospital (43 %), to stay at home (28 %) or to gain more physical power (9 %). There was no statistically significant difference to treatment goals documented for CA patients.

### Disease-related data

Patients with non-malignant illnesses most often suffered from diseases of the nervous (40 %), circulatory (18 %), genitourinary or respiratory (each 14 %) system (see Table 2).

**Table 2:** Primary non-cancer diagnosis (*N* = 87)

Primary non-cancer diagnosis		ICD-10	Percentage
Diseases of the	Nervous system (e. g. Morbus Parkinson)	F03, G00-G99	35 (40 %)
	Circulatory system (e. g. Heart Failure)	I00-I99	16 (19 %)
	Genitourinary system (e. g. UTI sepsis)	N00-N99	12 (14 %)
	Respiratory system (e. g. COPD)	J00-J99	12 (14 %)
	Digestive system (e. g. Liver Cirrhosis)	K00-K93	9 (10 %)
	Musculoskeletal system (e. g. Osteopathy)	M00-M99	2 (2 %)
	Infectious and parasitic diseases (e. g. HIV)	A00-B99	1 (1 %)
Overall			87 (100 %)

The most frequent NCA illnesses were dementia (18 %), liver cirrhosis (8 %) and multiple acute stroke syndrome (7 %).

NCA patients experienced by trend more often moderate or severe intensities of neurologic, psychiatric or psychological symptoms and problems (15 vs. 8 %), were in significantly higher need for assistance with defecation (87 vs. 74 %) and bladder function (47 vs. 29 %) and suffered considerably more often from moderate to severe deterioration of vigilance (somnolent or comatose) (30 vs. 11 %) as estimated in a symptom and problem checklist by professionals of the SPHC team (see Table 3).

**Table 3:** Group differences between non-cancer (*N* = 87) vs. cancer patients (*N* = 300) in percentage of patients suffering from moderate/severe intensities of certain symptoms and problems

Symptoms and problems	Non-cancer (*N* = 87)	Cancer (*N* = 300)	Level of significance
Pain	10 (12 %)	35 (12 %)	*p* = 1.00
Respiratory, cardiac	10 (12 %)	22 (7 %)	*p* = .246
Neurologic, psychiatric, psychological	13 (15 %)	24 (8 %)	*p* = .049
Gastrointestinal	7 (8 %)	33 (11 %)	*p* = .529
Urogenital	4 (5 %)	15 (5 %)	*p* = 1.00
Ulcerated wounds/ tumours	3 (3 %)	6 (2 %)	*p* = .419
Assistance with defecation	76 (87 %)	223 (74 %)	*p* = .001
Assistance with bladder function (permanent catheter)	41 (47 %)	88 (29 %)	*p* = .001
Deterioration of vigilance (somnolent/comatose)	26 (30 %)	34 (11 %)	*p* = .001
Social situation	8 (9 %)	16 (5 %)	*p* = .192
Ethical conflicts	5 (6 %)	15 (5 %)	*p* = .779
Problems in social law issues	3 (3 %)	8 (3 %)	*p* = .711
Burden of social support system	7 (8 %)	30 (10 %)	*p* = .670
Existential crisis	5 (6 %)	12 (4 %)	*p* = .542

### General patients’ condition and care-related data

At the time of admission to SPHC, significantly more NCA patients were classified to nursing care levels depending on individual needs for nursing support (61 vs. 31 %) as defined by the Medical Service of the Health Funds (Medizinischer Dienst der Krankenkassen, MDK) than CA patients.^1^ The main focus of support for the NCA patients was significantly more coordination of care (14 vs. 4 %) or counselling (15 vs. 2 %), but significantly less often partial care (e.g. in addition to nursing services, family physician etc.) (56 vs. 72 %) or complete care (full home care without involvement of other services) (13 vs. 20 %).

The mean number of face-to-face contacts to NCA patients was significantly lower (8 vs. 11 times) and the mean duration of care shorter (27 ± 53 vs. 31 ± 43 days; *p* = .454) than in CA patients. The percentage of NCA patients who died within the first 24 h after inclusion into SPHC was higher (12 vs. 5 %) than in CA patients. Less NCA than CA patients were re-admitted to hospital (6 vs. 20 %). The significant group differences in proportion of re-admittance is controlled by the patients’ age in logistic regression (*p* = .071). Significantly more NCA patients died in nursing homes (50 vs. 20 %), but less in private homes (30 vs. 55 %) than CA patients (see Table 4).

**Table 4:** Group differences between non-cancer (*N* = 87) vs. cancer patients (*N* = 300) in care-related information (significance level at *p* = 0.01)

		Non-cancer (*N* = 87)	Cancer (*N* = 300)	Level of significance
Nursing care level (need in assistance with body care, feeding, mobilization and housing)	None	33 (38 %)	201 (67 %)	*p* < .001
	1: 1.5 h per day	16 (18 %)	54 (18 %)	
	2: at least 3 h per day	19 (22 %)	31 (10 %)	
	3: at least 5 h per day	18 (21 %)	8 (3 %)	
	Missing Data	1 (1 %)	6 (2 %)	
Type of SPHC	Partial Care	49 (56 %)	215 (72 %)	*p* < .001
	Complete Care	11 (13 %)	61 (20 %)	
	Coordination	12 (14 %)	11 (4 %)	
	Counselling	13 (15 %)	6 (2 %)	
	Missing Data	2 (2 %)	7 (2 %)	
Number of involved professional caregiver		1.2 ± 0.4	1.4 ± 0.6	*p* = .068
Number of general medical equipment provided (e.g. wheel chair)		3.4 ± 2.0	2.9 ± 1.8	*p* = .084
Duration of SPHC		27 ± 53 days	31 ± 43 days	*p* = .454
Number of SPHC visits		8 ± 8 visits	11 ± 9 visits	*p* = .011
Death < 1 day		10 (12 %)	15 (5 %)	*p* = .044
Re-admittance to hospital		6 (6 %)	60 (20 %)	*p* < .001
		1.3 ± 0.5 times	1.5 ± 1.4 times	*p* = .723
		11 ± 8 days	11 ± 13 days	*p* = .889
End of SPHC	Discharge from SPHC	17 (20 %)	31 (10 %)	*p* = .027
	Death	70 (80 %)	269 (90 %)	
Place of death *N* = 339 (70/269)	At Home	21 (30 %)	149 (55 %)	*p* < .001
	Nursing Home	35 (50 %)	53 (20 %)	
	Hospice	10 (15 %)	44 (16 %)	
	Hospital	2 (3 %)	18 (7 %)	
	Other Institutions	1 (1 %)	1 (0 %)	
	Missing Data	1 (1 %)	4 (2 %)	

A higher proportion of NCA patients (20 vs. 10 %) was discharged from SPHC care.

## Discussion

In Germany little is known about specialized PC at home for terminally ill patients suffering from non-malignant diseases. To the best of our knowledge, this is the first study comparing SPHC care for terminally ill NCA to CA patients. The central findings from this investigation point at disadvantageous differences of NCAs versus CAs regarding age, marital status and living situation, nursing care needs and restricted abilities for everyday life, the type of SPHC offered, re-admittance to hospital and place of death which is comparable to international research results [19].

The study subject shows the importance of taking into account the specific needs and characteristics of patients with NCA diseases in specialized palliative home care. Beside the distinction between CA and NCA patients, the results underline the influence of individual parameters such as the patient’s age, family status, living situation, symptoms and problems and time of access to SPHC.

The results of the investigation presented here show that end-of-life needs for PC in NCs are at least as complex and intense as they are in CAs [20]. The need for nursing assistance, medical equipment and the burden with symptoms are at a comparable level with CAs. The authors conclude that the integration of palliative home care takes place rather late in the disease trajectory of both NCAs and CAs as seen in the very short survival time for a considerable part of both patient groups. Therefore, the expansion of specialized PC structures and opportunities which meet the special needs of NCAs is urgently needed.

In both patient groups, the overall number of patients who were re-admitted to hospital was low. This may, on the one hand, be due to the frequent patient wishes to avoid re-admittance to hospital and to remain at home. On the other hand, these goals may also be encouraged by policy makers and health care insurances who intended SPHC teams to keep patients stable at home, while saving costs on expensive, long-term hospital stays [1, 12].

In Germany the access for NCA patients to palliative home care is still hampered. Two main reasons may be mentioned here. The national report of an evaluation of the permission of PC activities by German health care insurances shows that the coverage of costs for specialised home care PC was denied especially (I) if patients were privately insured or (II) were suffering from a non-malignant disease [6]. This hypothesis is reinforced by the fact that a higher proportion of NCA patients is, following primary inclusion, again discharged alive from SPHC, perhaps due to possible retraction of consent for reimbursement by their health care insurance. These aspects may at least in part help explain why we have not reached an equal distribution of diagnosis groups in palliative and hospice care services although this 50:50 distribution is expected for Europe in the upcoming years. Several barriers and challenges why non-cancer patients are under-represented on the caseloads of community Macmillan specialist palliative care nurses were also reported by Andrews and colleagues (2011). The authors argue that especially the viewpoints of patients and carers should be considered and new innovative models of service delivery be developed [21].

The local situation found in our study is congruent to the development in other countries such as the UK. For the UK the National Survey of Patient Activity Data for Specialist Palliative Care Services from 2010–2011 summarizes that there, as well, only 21 % of all home care patients had a primary NCA diagnosis [22], with a similar distribution for circulatory, respiratory and neurological diseases.

Steps in the right direction have been made and positive trends can be seen in the development of PC internationally [23], but still a lot of work and initiatives are needed to influence policy and develop adequate long-term financing structures for NCA patients with PC needs [24].

### Study limitations

Due to the study design of a retrospective data analysis, only routine data from clinical practice were investigated here. Therefore, the results have to be interpreted cautiously. The analysis has a strongly explorative and descriptive character due to the novelty of the service implementation and provides only basic data. In addition, the subgroup of NCA and CA are heterogeneous in itself and include different primary diseases which may have contributed to differences in needs and symptoms. Future research should conduct a prospective study including the involvement of the patients themselves in data collection.

Due to changes in documentation patterns, systems and items during the investigated period, a considerable percentage of missing data in several variables occurred. No validated symptom assessment tool was used, hence limiting, to a certain extent, the value of data on symptoms and problems.

As SPHC teams in Germany are individually organized and structured, they are free and differ in their documentation routines and priorities, so that the findings of a single service is not representative and therefore cannot be generalized to other regions, SPHC teams and patient groups.

## Conclusions

Main findings indicate that the proportion of NCA patients among all patients receiving SPHC was high in the investigated single service, but in accordance to an overall assessments of SPHC in Bavaria and therefore rather representative [18]. They have severe PC needs but relatively late access to PC services.

Although more and more patients suffering from non-malignant diseases are equally in need of specialist PC as CA patients and are slowly getting access to PC and hospice services, financing models are still not adequate. More political developments are needed to fill this gap.

## Abbreviations

CA: Cancer
ISPC®: Information System Palliative Care 3.0
NCA: Non-cancer
PC: Palliative care
SPHC: Specialized Palliative Home Care
WHO: World Health Organization

## Footnotes

1 In Germany, an application to determine the need for nursing care for a certain patient is made to the Medical Service of the Health Funds (MDK). This application is followed by a face-to-face meeting between the certain patient and an employee of the MDK. This employee is authorized to evaluate the individual need for nursing care e.g. in body care, mobility, management of one’s own household. The need for nursing care in these tasks corresponds to defined time units. The sum of all time units then results in a proposal of the employee for a nursing care level to the Health Funds.
